# Neutralizing Antibodies Response against SARS-CoV-2 Variants of Concern Elicited by Prior Infection or mRNA BNT162b2 Vaccination

**DOI:** 10.3390/vaccines10060874

**Published:** 2022-05-30

**Authors:** Floriana Bonura, Dario Genovese, Emanuele Amodio, Giuseppe Calamusa, Giuseppa Luisa Sanfilippo, Federica Cacioppo, Giovanni Maurizio Giammanco, Simona De Grazia, Donatella Ferraro

**Affiliations:** Department of Health Promotion Sciences, Maternal and Infant Care, Internal Medicine and Medical Specialties “G. D’Alessandro”, University of Palermo, Via Del Vespro 133, 90127 Palermo, Italy; floriana.bonura82@gmail.com (F.B.); dario.genovese@unipa.it (D.G.); giuseppe.calamusa@unipa.it (G.C.); sanfilippogiusi@gmail.com (G.L.S.); federica-cacioppo@libero.it (F.C.); giovanni.giammanco@unipa.it (G.M.G.); simona.degrazia@unipa.it (S.D.G.); donatella.ferraro@unipa.it (D.F.)

**Keywords:** SARS-CoV-2, VOC, Omicron, Italy, neutralizing antibody titers, NtAb

## Abstract

In order to determine the humoral protective response against SARS-CoV-2, the vaccine-induced and naturally induced neutralizing antibodies (NtAbs) responses against SARS-CoV-2 variants circulating in Italy through in vitro live virus neutralization assay were evaluated. A total of 39 SARS-CoV-2 recovered subjects (COVID-19+) and 63 subjects with a two-dose cycle of the BNT16262 vaccine were enrolled. A single serum sample was tested for COVID-19+ at 35–52 days post-positive swab, while vaccinees blood samples were taken at one (V1) and at three months (V3) after administration of the second vaccine dose. Significantly higher NtAb titers were found against B.1 and Alpha in both COVID-19+ and vaccinees, while lower NtAb titers were detected against Delta, Gamma, and Omicron variants. A comparison between groups showed that NtAb titers were significantly higher in both V1 and V3 than in COVID-19+, except against the Omicron variant where no significant difference was found. COVID-19+ showed lower neutralizing titers against all viral variants when compared to the vaccinees. Two-dose vaccination induced a sustained antibody response against each analyzed variant, except for Omicron. The evolution process of SARS-CoV-2, through variants originating from an accumulation of mutations, can erode the neutralizing effectiveness of natural and vaccine-elicited immunity. Therefore, a need for new vaccines should be evaluated to contain the ongoing pandemic.

## 1. Introduction

The pandemic spread of SARS-CoV-2 [1] has resulted in the selection of a consistent number of variants or genetic lineages. Some of these variants or groups of them were defined as Variants of Concern (VOC) due to increased transmissibility and virulence (severity) and/or their ability to evade immunity [2,3]. To help track the transmission and spread of SARS-CoV-2 genetic lineages, a dynamic nomenclature system called the PANGO network was proposed in April 2020 [4]. As of 31 May 2021, the World Health Organization (WHO) proposed the use of letters of the Greek Alphabet in communications about variants to the public [2].

In Italy, the first documented case of SARS-CoV-2 infection dates back to 20 February 2020: a 38-year-old male patient from Codogno (Lodi, Italy) [5]. During the initial months of the pandemic, the most frequently detected strains in Italy were PANGO lineages B.1 and B.1.1.74 [6], both belonging to the first haplotype discovered, lineage B [7].

The PANGO lineage B.1.1.7, then renamed the Alpha variant, was first detected in the United Kingdom on 20 September 2020 and by February 2021 accounted for approximately 95% of the viral transmission in the UK [8].

In Italy, the Alpha variant became the prevalent lineage (54% of typed samples) starting from 18 February 2021, according to the surveillance reports of the Italian Institute of Health (Epicentro, ISS) [9]. This strain is characterized by nine mutations in the Spike protein of SARS-CoV-2, including the N501Y mutation and the deletion of two amino acids at positions 69 and 70 [10], and an estimated 43 to 90% higher reproduction number than the previously circulating lineages [8], with a higher hospitalization and mortality rate.

The Delta variant (PANGO lineage B.1.617.2, sub-lineage of B.1.617) was first detected in India in October 2020. Due to its increased transmissibility and higher risk of hospital admission and emergency care attendance compared to the Alpha variant [11,12], on 11 May 2021, the WHO designated Delta as a VOC. The B.1.617.2 variant is characterized by several Spike protein mutations, including L452R and P681R, that apparently affect the immune responses, reducing the COVID-19 vaccines’ efficacy against this variant in terms of symptomatic disease [13].

The Gamma variant, identified in Brazil in October 2020, in the Manaus area [14], despite originating from the B strain, has been shown to be phylogenetically different from previous variants and has therefore received a new haplotypic designation, PANGO lineage P.1. The Gamma variant is characterized primarily by three Spike protein mutations, E484K, N501Y, and K417T: these mutations have been linked to a higher transmission rate [15].

The Omicron strain (PANGO lineage B.1.1.529) was first identified in South Africa and Botswana in November 2021. This novel variant owns a large number of mutations with immunoevasive potential, justifying its rapid spread worldwide and an increased risk of reinfection compared to previous VOCs [16].

Anti-COVID-19 vaccines have been developed to prevent the recurrence of COVID-19 pandemic waves. BNT162b2 was one of the first mRNA vaccines ever developed. It works by the stimulation of the immune system to produce specific antibodies to the SARS-CoV-2 Spike protein, using lipid nanoparticles-embedded mRNA encoding for the SARS-CoV-2 full-length Spike protein derived from the first SARS-CoV-2 documented virus, named the “WH-Human 1” coronavirus. Although this vaccine was initially highly effective against COVID-19 illness and hospitalizations [17,18,19], changes in the coding sequence of the Spike protein among different variants could adversely affect the vaccine-induced immune response, making the vaccine less effective or even ineffective.

Therefore, the initial two-dose BNT-162b2 schedule has been modified introducing a 3rd injection to boost the Spike-specific immunity [20,21].

In this study, an in vitro neutralization assay was performed in order to comprehensively estimate vaccine-induced and naturally induced neutralizing antibody levels against SARS-CoV-2 variants circulating in Italy, enrolling SARS-CoV-2 recovered subjects and subjects who had received two doses of the BNT162b2 vaccine.

## 2. Materials and Methods

### 2.1. Study Population

A total of 102 healthcare workers (HCWs) at the A.O.U.P. “P. Giaccone” Hospital of Palermo (Italy) were enrolled in the study between 15 January 2021 and 15 February 2021. Of these, 39 subjects—henceforth named COVID-19+—had recovered from previous SARS-CoV-2 infection, while the remaining 63 subjects had been vaccinated with two doses of Comirnaty (BNT162b2) Pfizer-BioNTech mRNA COVID-19 vaccine [17,22].

The following inclusion criteria were used: (a) for COVID-19+ patients, a previous SARS-CoV-2 infection certified by a positive nasopharyngeal swab (NPS) recorded at the Virology Laboratory of A.O.U.P. “P. Giaccone” Hospital; leftover NPS samples were properly stored for biomolecular analysis and serum samples taken at least 20 days after the first positive NPS were available; (b) for vaccinated subjects, being anti-S IgG negative at the time of administration of the first vaccine dose, accessing the first dose of BNT162b2 vaccine, and receiving the second dose within scheduled time frame (21 to 28 days from first dose).

All vaccinees had been vaccinated according to Pfizer/BioNTech manufacturer instruction and vaccine administration had been performed according to the guidelines provided by the Italian Medicines Agency—AIFA. Enrolled vaccinated subjects underwent two blood samplings: at one and three months (V1 and V3 sera) after completion of the two-dose vaccination cycle. A single serum sample was evaluated for COVID-19+ subjects. Demographic data (sex and age) were collected for all 102 HCW participants, and date of first and second dose vaccination were recorded for vaccinees.

### 2.2. Quantitative Determination of anti-SARS-CoV-2 S1/S2 IgG

Serum specimens collected from each subject were tested using a quantitative assay LIAISON^®^ SARS-CoV-2 S1/S2 IgG (DiaSorin S.p.A., Saluggia, Italy) to evaluate the presence of IgG antibodies against SARS-CoV-2 S1/S2 antigens, according to the manufacturer’s instructions. The IgG antibody levels >15 AU/mL (Arbitrary Units/mL) were considered positive; conversely, IgG antibody levels <12 AU/mL were negative. IgG levels ranging from 12 to 15 were considered as borderline.

### 2.3. Neutralizing Antibodies

We used a previously described in vitro live virus-neutralization assay to quantify neutralizing antibodies against replication competent B.1, Alpha, Delta, Gamma, and Omicron strains of SARS-CoV-2 obtained from clinical samples [23]. All live virus micro-neutralization assays were performed in the BioSafety Level (BSL)-3 laboratory. Testing procedures of clinical specimens were carried out through strict observance of the WHO interim guidance [24]. Briefly, the titers of neutralizing antibodies (NtAbs) were determined using Vero E6 cells separately infected with B.1, Alpha, Delta, Gamma, and Omicron SARS-CoV-2 variants. NtAbs titers were defined as the reciprocal value of the sample dilution that showed a 50% protection of virus-induced cytopathic effect (ID${}_{50}$). Titers below 10 were regarded as negative.

### 2.4. Genotyping of Virus Variants

The genetic characteristics of the SARS-CoV-2 viral variants in NPSs of COVID-19+ patients were assessed by SARS-CoV-2 Variants ELITe MGB® Kit (ELITechGroup S.p.A, Torino, Italy) through Reverse Transcription Real-Time Polymerase Chain Reaction and melting curve analysis of the mutations of the Spike protein (S) gene. Next Generation Sequencing (NGS) technology analysis of SARS-CoV-2 variants’ S gene, using a MiSeq device (Illumina Inc., San Diego, CA, USA), was used to ensure genetic characteristics, and genetic stability after virus replication in cell culture was performed for neutralization tests.

### 2.5. Statistical Analyses

Qualitative data have been summarized by frequency and relative frequencies (%), whereas quantitative variables have been shown as mean (standard deviation—SD) if normally distributed and median (interquartile range—IQR) for not normally distributed variables.

Shapiro–Wilk test has been used to verify normal distribution of quantitative variables.

Pearson’s Chi-squared test was used to assess whether samples frequencies were different or not, while Mann–Whitney–Wilcoxon test was used to evaluate differences of sample distributions between groups.

All statistical analyses were conducted using the R for Statistical Computing program (R version 4.0.5, Vienna, Austria) and a *p*-value <0.05 was considered as statistically significant [25].

## 3. Results

The general characteristics of the COVID-19+ subjects and vaccinees at 1 month (V1) and at 3 months (V3) are reported in Table 1. The differences observed amongst the COVID-19+ and vaccinated subjects for gender distribution (64.1% of females in COVID-19+ group vs. 58.7% of females in vaccinated group; *p*-value = 0.74) and age (47 years old in COVID-19+ group vs. 52 years old in vaccinated group; *p*-value = 0.91) were not statistically significant. The analysis of the mutations of the S gene allowed to ascertain that all COVID-19+ subjects had been infected by the B.1 strain. The median distance between the 1st dose of the vaccine or SARS-CoV-2 positivity and blood sampling was 44 days among the COVID-19+ group, 29 days in the V1 group, and 112 days in the V3 group.

**Figure 1:**
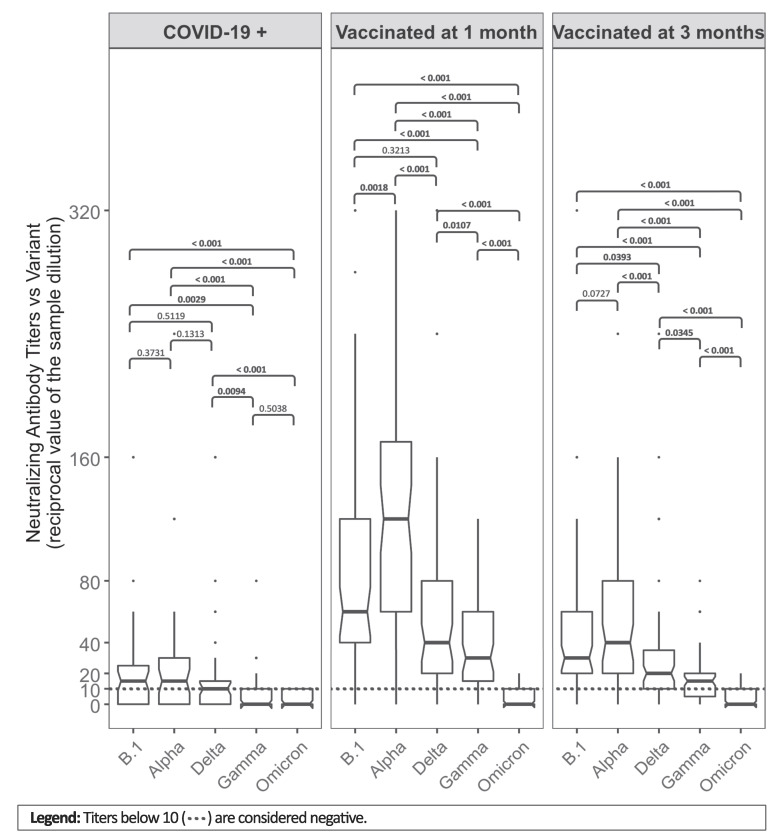
Comparison between median (IQR) neutralizing antibody titers against different variants within COVID-19+ subjects, vaccinated at 1 and 3 months groups (V1 and V3), using Wilcoxon test with its relative *p*-values. Titers below 10 (dotted lines) are considered negative.

In Figure 1, the NtAb titers against different VOCs (B.1, Alpha, Delta, Gamma, and Omicron variants) are compared within each group (COVID-19+, V1, and V3). In all groups, higher NtAb titers were found against B.1 and Alpha, being lower against Delta and Gamma, while antibodies against the Omicron variant mostly did not reach the threshold for positive results (titer below 10).

An absence of NtAbs activity was observed among COVID-19+ subjects in 11 (28.2%) serum samples against the Alpha variant and in 27 (69.2%) against the Omicron variant. Amongst the V1 sera, only 1 sample (1.6%) lacked antibodies against the Alpha strain, and 37 (58.7%) against the Omicron strain. After 3 months from vaccine administration, 5 subjects (7.9%) did not show NtAbs against the Alpha variant and 46 (73%) against the Omicron strain.

For each of the three groups, almost all distributions were statistically significantly different from the others.

**Figure 2:**
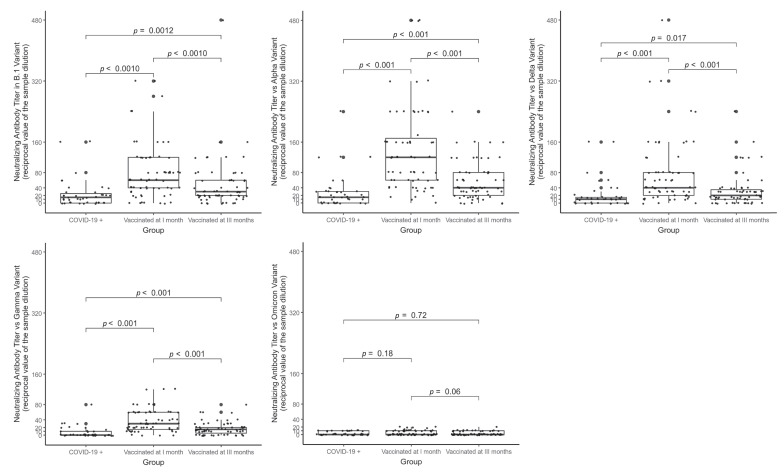
Comparison between median (IQR) neutralizing antibody titers against different variants among COVID-19+ subjects and Vaccinees at 1 month and 3 months (Wilcoxon test with its relative *p*-values are shown in the table).

Figure 2 graphically illustrates the significance of the differences in the NtAb titers observed when the sera from each group were tested against different viral variants (B.1, Alpha, Delta, Gamma, and Omicron variants). Overall, the NtAb titers in both the V1 and V3 were statistically significantly higher than in the COVID-19+, except for the Omicron strain neutralization where no significant difference was found between the three groups. For the other variants, differences in neutralizing titers resulted to be statistically significant, with *p*-values ranging from *p* = 0.02 to *p* < 0.001.

## 4. Discussion

Immunity to SARS-CoV-2, induced by vaccination or by natural infection, has been shown to reduce the risk of clinically significant outcomes [17,26]. NtAbs play a crucial role in binding the SARS-CoV-2 Spike protein, preventing it to mediate host cell entry. The evaluation of NtAb titers is useful to determine vaccine efficacy and humoral protective response longevity. Nowadays, a few mRNA- and vector-based vaccines have been developed using the Spike glycoprotein belonging to “WH-Human 1” SARS-CoV-2 and are offered to provide protective immunity. A critical challenge for long-term vaccine efficacy is represented by the emergence of several variants of the virus that harbor mutations in the major target for NtAbs, the Receptor Binding Domain (RBD). The mutations in the RBD may cause a rapid decline of the NtAbs and affect the efficacy of vaccines and the protective responses against reinfection. The literature data show that the Alpha, Delta, and Gamma variants might not differ markedly in host–cell interactions, while major differences in susceptibility to antibody-mediated neutralization were observed [27,28]. Conversely, the rapid global spread of the Omicron variant strongly suggested a more efficient cell entry mechanism and virus production, resulting in increased infectivity and transmission, other than a low-to-absent neutralizing response in vitro to previous VOC immunity [29].

According to this intriguing question, this study reports the neutralizing response of SARS-CoV-2-recovered subjects and vaccinees against the epidemiologically relevant variants that have been circulating in Italy since the beginning of the SARS-CoV-2 pandemic.

Within the three different groups of the subjects analyzed (COVID-19+, V1, and V3), we have observed interesting differences in the levels of NtAbs toward the five SARS-CoV-2 strains tested (ancestor B.1, and the Alpha, Delta, Gamma, and Omicron variants).

Although the group of COVID-19+ subjects had statistically significantly lower median neutralizing antibody titers compared to vaccinated subjects, regardless of the month post-vaccination, both infected and vaccinees groups showed very low or nil neutralizing antibody levels against the Omicron strain. In fact, the Omicron variant shows as much as 59 mutations in the Spike protein but shares with the Gamma and Delta variants only 3 of its 15 mutations within the RBD (K417, E484, and N501). The abundance of mutations in the Spike of the Omicron variant results in a less effective neutralizing response in the sera from vaccinees [16,30,31,32].

When testing the sera on the remaining SARS-CoV-2 variants, robust NtAb titers were observed against the B.1 and Alpha strains in all the study groups; conversely, lower neutralization titers were observed against the Delta and Gamma variants. As expected, the COVID-19+ subjects had higher NtAb titers against the ancestor B.1 strain that had caused their infection, but similar NtAb levels were also detected against the Alpha variant.

These results are confirmed by many studies that showed a substantial reduction in NtAb titers elicited by natural infection or complete-cycle vaccination toward the Delta and Gamma variants compared to B.1 and Alpha [27,28,33,34,35]. Significantly decreased neutralization titers against the Omicron variant in vaccinees were also previously observed [32]. The finding of decreased neutralizing immunity against subsequent variants after natural infection may explain the recently reported rise in cases of reinfection with newer variants [36,37,38,39]. It is interesting to note that median NtAb titers in all groups were higher against the Alpha variant than against the B.1 strain. This suggests that both natural and artificial immunity could be more protective toward the Alpha strain than toward the first variant circulating in Italy. These results seem to be confirmed by several studies stating that 6 weeks after the administration of the 2nd dose of the vaccine, 92% of the samples showed an efficient neutralizing activity toward the B.1 and Alpha variants [40]. In reverse, a study conducted by Muik et al. [41] on a group of two-dose vaccinees, evidenced slight but statistically significantly reduced NtAb titers against the Alpha variant compared with the B.1 Wuhan virus reference. Nevertheless, a slight reduction in titer should not be a biologically relevant change in terms of the neutralization efficacy.

The findings related to the Gamma variant were of concern: the ability to produce neutralizing antibodies against this variant was low in all three groups. These results are confirmed by Lustig et al. [42] and especially by Hoffmann et al. [27] who found that 12 out of 15 sera had a marked reduction in the neutralization of the Gamma variant Spike protein, while all sera efficiently neutralized the Wild-Type Spike protein.

The absence of NtAbs against the Omicron variant in all of the three groups suggests an almost total evasion of the Omicron variant from Spike protein-specific immunity, regardless of whether it is derived from a natural infection or from a two-dose vaccination course. Several studies are in agreement with our findings, showing an extensive reduction in NtAb titers against the Omicron strain compared to the Wuhan ancestor [29,43,44].

Although no threshold for protection has been defined, the absence of NtAbs against the Gamma and Omicron variants in two-thirds of COVID-19+ subjects suggests that antibody production deriving from natural infection could be ineffective in a relevant part of recovered patients, unlike the protection provided by vaccination that seems to remain effective for a short time after two doses and longer after a 3rd booster vaccination [20]. However, during natural infection, cellular immunity can mitigate the severity of illness even in the absence of NtAbs [45]. The COVID-19+ subjects showed lower neutralizing titers against all viral variants when compared to the vaccinees, and the difference was strongly statistically significant. This result strengthens the need to support vaccination campaigns aimed at achieving at least 90% coverage and stresses the usefulness of a third vaccine dose.

The present study has several limitations. Synthetically, these limitations are: (i) a relatively small number of participants, despite the significancy of the findings; (ii) considering the in vitro results, these need to be confirmed in vivo as to whether the NtAb titer correlates with protection. In fact, real-world vaccine effectiveness can be modified by multiple factors, such as comorbidities and other individual differences influencing the waning of immunity [46] or favoring a long-lived humoral immunity [47,48]. Moreover, further affinity maturation of antibodies must be taken into account as, with aging, humoral immunity could be able to cross-recognize novel epitopes appearing in emerging variants; (iii) the lower neutralizing response in COVID-19+ subjects compared to vaccinees might have been influenced by differences in the exposure to the immunogenic target as, during infection, immunity is stimulated by the entire antigenic viral repertoire, while only the Spike protein is used as an immunological target in the anti-COVID-19 vaccines; (iv) in regard to the vaccinated subjects group, they have been evaluated as only two-dose vaccinees and, therefore, further studies will be necessary to understand the role of a third dose of BNT162b2 against the different viral variants, including the Omicron strain. In recent studies, a cross-reactivity enhancement of the NtAbs response allowing to neutralize the Omicron variant has been observed after a third booster dose of vaccine [43,49].

Overall, the results of this study suggest that the BNT162b2 vaccination induces a sustained antibody response against each of the variants analyzed, except for the Omicron strain, and serological responses are higher than those observed in COVID-19+ subjects.

The continuing evolution process of the SARS-CoV-2 variants, due to a constant accumulation of mutations, can erode the neutralizing effectiveness of natural and vaccine-elicited immunity.

Therefore, continuous epidemiological and immunological surveillance will be crucial to evaluate the need for new vaccines and to contain the ongoing pandemic, because a virus will only spread in a community when it finds no or insufficient immunity.

## Figures and Tables

**Table 1:** Demographic and serological characteristics of the three groups studied: COVID-19+ subjects and vaccinees at 1 month (V1) and at 3 months (V3) from 1st vaccine dose.

		Groups, Median (IQR)	
	**COVID-19 +**	**Vaccinated at 1 Month**	**Vaccinated at 3 Months**
	**(N = 39)**	**(N = 63)**	**(N = 63)**
*Sex, N (%)*			
- Males	14 (35.9%)	26 (41.3%)	26 (41.3%)
- Females	25 (64.1%)	37 (58.7%)	37 (58.7%)
*Age in years*	47 (36–57)	52 (39.5–55)	52 (39.5–55)
*1st dose/COVID+ blood sample, gap in days*	44 (35–52)	29 (28–34)	112 (97–116.2)
*Antibody titers—total IgG (AU/mL)*	140.75 (78.25–322.50) ^1^	1002 (660–4215) ^2^	570 (351–777) ^2^
*Neutralizing antibody titers against variants (reciprocal value of the sample dilution)*			
- B.1	15 (0–25)	60 (40–120)	30 (20–60)
- Alpha	15 (0–30)	120 (60–170)	40 (20–80)
- Delta	10 (0–17.5)	40 (20–80)	20 (10–35)
- Gamma	0 (0–10)	30 (15–60)	15 (5–20)
- Omicron	0 (0–10)	0 (0–10)	0 (0–10)
*Absence of neutralizing antibody titers against variants, N (%)*			
- B.1	13 (33.3%)	7 (11.1%)	8 (12.7%)
- Alpha	11 (28.2%)	1 (1.6%)	5 (7.9%)
- Delta	13 (33.3%)	3 (4.8%)	13 (20.6%)
- Gamma	26 (66.7%)	4 (6.3%)	16 (25.4%)
- Omicron	27 (69.2%)	37 (58.7%)	46 (73%)

^1^ Antibody titers are referred to blood withdrawal taken after COVID-19 diagnosis for COVID-19+ subjects. ^2^ Antibody titers are referred to blood withdrawal taken at 1 month or at 3 months after 1st dose administration for Vaccinated at 1 month and Vaccinated at 3 months, respectively.

## Data Availability

The dataset generated during and/or analyzed during the current study is not publicly available due to privacy reasons but is available from the corresponding author on reasonable request.
